# A novel transcription factor gene *FHS1* is involved in the DNA damage response in *Fusarium graminearum*

**DOI:** 10.1038/srep21572

**Published:** 2016-02-18

**Authors:** Hokyoung Son, Minmin Fu, Yoonji Lee, Jae Yun Lim, Kyunghun Min, Jin-Cheol Kim, Gyung Ja Choi, Yin-Won Lee

**Affiliations:** 1 Department of Agricultural Biotechnology and Centre for Fungal Pathogenesis, Seoul National University, Seoul 08826, Republic of Korea; 2 Centre for Food and Bioconvergence, Seoul National University, Seoul 08826, Republic of Korea; 3 Division of Applied Bioscience and Biotechnology, Institute of Environmentally Friendly Agriculture, College of Agriculture and Life Sciences, Chonnam National University, Gwangju 61186, Republic of Korea; 4 Eco-friendly New Materials Research Group, Research Centre for Biobased Chemistry, Division of Convergence Chemistry, Korea Research Institute of Chemical Technology, Daejeon 34114, Republic of Korea

## Abstract

Cell cycle regulation and the maintenance of genome integrity are crucial for the development and virulence of the pathogenic plant fungus *Fusarium graminearum*. To identify transcription factors (TFs) related to these processes, four DNA-damaging agents were applied to screen a *F. graminearum* TF mutant library. Sixteen TFs were identified to be likely involved in DNA damage responses. Fhs1 is a fungal specific Zn(II)_2_Cys_6_ TF that localises exclusively to nuclei. *fhs1* deletion mutants were hypersensitive to hydroxyurea and defective in mitotic cell division. Moreover, deletion of *FHS1* resulted in defects in perithecia production and virulence and led to the accumulation of DNA damage. Our genetic evidence demonstrated that the *FHS1*-associated signalling pathway for DNA damage response is independent of the ATM or ATR pathways. This study identified sixteen genes involved in the DNA damage response and is the first to characterise the novel transcription factor gene *FHS1*, which is involved in the DNA damage response. The results provide new insights into mechanisms underlying DNA damage responses in fungi, including *F. graminearum*.

DNA functions as the carrier of genetic information, and therefore genome integrity and stability are essential for all organisms. However, DNA is easily subjected to damage from both intrinsic and extrinsic sources, including single- and double-strand breaks, base damage, and DNA-protein crosslinks^1^. If left unrepaired or incorrectly repaired, damaged DNA will result in cell cycle arrest, cell death, loss of genetic information, and genomic instability^2^.

To address the fundamental problem of genomic erosion, DNA damage response (DDR) systems have evolved to detect DNA damage and promote the repair of damaged DNA. DDR ensures the maintenance of genome integrity and stability by regulating DNA repair mechanisms. Moreover, DNA replication, gene transcription, DNA repair, and cell cycle checkpoints should be interlinked to assure cell survival and the normal regulation of cellular functions following DNA damage^3^,^4^.

Despite significant advances in agricultural biotechnology and fungal genetics, fungal threats to public health and food security are still increasing^5^. *Fusarium graminearum* is a disastrous pathogenic plant fungus and is the major causative agent of *Fusarium* head blight (FHB) on major cereal crops worldwide^6^. *F. graminearum* causes yield and quality losses due to sterility of florets and formation of discoloured, withered and light test weight kernels^7^. In addition, the fungal infection results in the accumulation of mycotoxins, such as zearalenone and deoxynivalenol, that are harmful to animals and humans^8^. Because several previously proposed strategies to control FHB, including biological and chemical measures, have not been effective in preventing disease outbreaks, FHB epidemics continue to occur throughout the world^7^.

Various forward and reverse genetic approaches have been applied in *F. graminearum*; however, most of the previous studies have focused on signal transduction pathways or orthologous genes, which tend to be highly conserved in eukaryotes^7^,^9^,^10^. Characterisation of novel genes or mechanisms involved in central transduction pathways or tolerance to specific environmental stresses would be novel molecular targets for the development of new chemical/biological pesticides or molecular breeding. Massive genetic approaches applying forward and reverse genetic tools are necessary for mining those genes or mechanisms.

In a previous study, we constructed a *F. graminearum* transcription factor (TF) gene deletion mutant library and obtained a comprehensive phenotypic dataset, which can be accessed at FgTFPD (http://ftfd.snu.ac.kr/FgTFPD)^11^. The results identified TFs involved in sexual and asexual reproduction, secondary metabolism, stress responses, and virulence. Because TFs are involved in most biological processes, screening the TF mutant library with specific stress conditions will shed light on novel mechanisms for these processes. As mentioned above, all organisms encounter DNA damage and have evolved their own DNA repair mechanisms. Therefore, the identification of TFs related to DNA damage responses could uncover novel mechanisms in *F. graminearum*.

The objectives for this study were to 1) construct a phenotypic dataset for DNA damage responses from the *F. graminearum* TF mutant library and 2) understand the molecular mechanisms underlying the DNA damage responses of a novel TF in *F. graminearum*. We screened the previously generated *F. graminearum* TF mutant library using four different DNA-damaging agents and discovered that the *fhs1* mutant was highly sensitive to hydroxyurea (HU). To elucidate the regulatory mechanisms for the hypersensitivity of the *fhs1* mutant to HU, we performed genetic studies and analysed the transcriptome. This study is the first to characterise a novel transcription factor in DNA damage responses and will provide new insights into mechanisms underlying the DNA damage responses in *F. graminearum* as well as other filamentous fungi.

## Results

### Identification of transcription factors involved in DNA damage responses

To identify the transcription factors involved in DNA damage responses, we screened 675 previously generated^11^ transcription factor gene deletion mutants using four chemical reagents that cause different types of DNA damage (Supplementary Table S1). Experimental conditions were established such that the *F. graminearum* wild-type strain exhibited approximately one half of the radial growth observed on complete medium (CM) without any DNA-damaging agent: methyl methanesulfonate (MMS, 0.1 μl/ml), hydroxyurea (HU, 10 mM), bleomycin (BLM, 10 mU/ml), and camptothecin (CPT, 0.4 μM).

**Figure 1:**
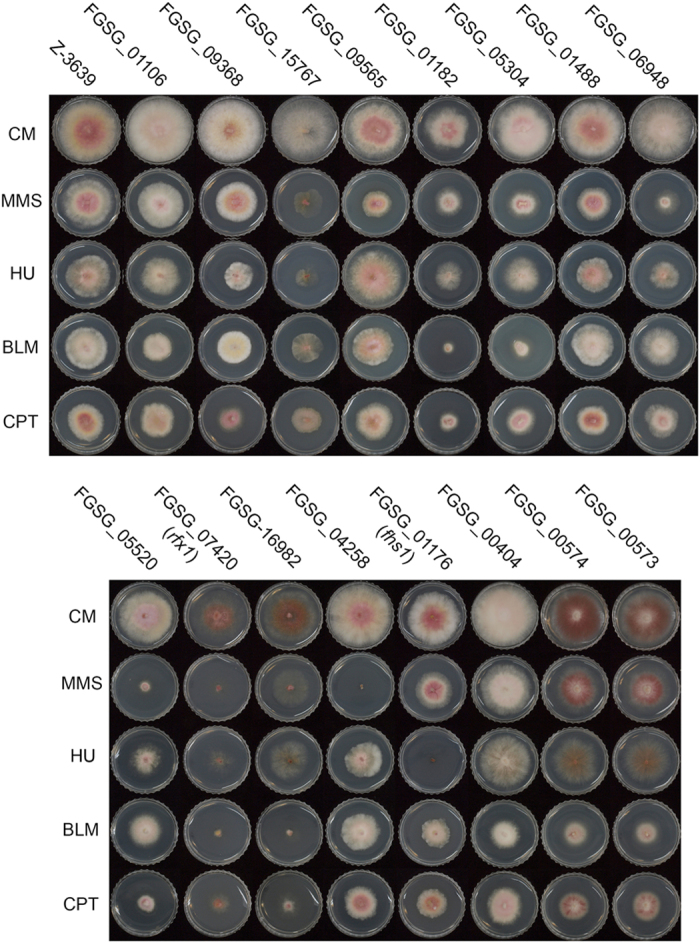
Sixteen transcription factor gene deletion mutants exhibited altered sensitivity to DNA-damaging agents. Pictures were taken 3 days after inoculation on CM with or without DNA-damaging agent (0.1 μl/ml MMS [methyl methanesulfonate], 10 mM HU [hydroxyurea], 10 mU/ml BLM [bleomycin], and 0.4 μM CPT [camptothecin]). Each locus ID is denoted above the corresponding deletion mutant.

Sixteen mutants exhibited altered sensitivity to at least one DNA-damaging agent (Table 1 and Fig. 1). Some of the screening results were consistent with previous studies. *RFX1* functions in DNA damage repair in *F. graminearum*^12^. Homologues of *ARS2* (FGSG_01106)^13^, *MSN2* (FGSG_09368)^14^, *SFP1* (FGSG_15767)^15^, *CTR9* (FGSG_06948)^16^, *CUL-4A* (FGSG_05520)^17^, *MYO1* (FGSG_16982)^18^, and *RAD18* (FGSG_04258)^19^ are all required for DNA damage responses and/or cytokinesis in model organisms.

Homologues of *DBP3* (FGSG_05304) and *DBP4* (FGSG_01182) encode the third and fourth subunits of DNA polymerase ε, respectively^20^,^21^, and both FGSG_00573 and FGSG_00574 are predicted to encode Ppr1, which regulates *de novo* pyrimidine biosynthesis in yeast^22^. These four genes are potentially involved in DNA damage responses, because they are closely linked to DNA replication and nucleotide synthesis^1^.

FGSG_09565 encodes a homologue of the GATA transcription factor SreA, which is involved in siderophore biosynthesis in *A. nidulans*^23^. A putative transcription factor containing an HMG box DNA-binding domain (FGSG_01488) and two genes (FGSG_01176 and FGSG_00404) with a Zn(II)_2_Cys_6_ fungal-type DNA-binding domain (IPR001138) have not been functionally characterised in other fungi. Of these, we selected one mutant (FGSG_01176) that was specifically sensitive to HU for further study and designated the gene name *F. graminearum* Hydroxyurea Sensitive 1 (*FHS1*).

### Identification of the novel transcription factor gene *FHS1* in *F. graminearum*

*FHS1* (locus ID: FGSG_01176) encodes 492 amino acids containing the Zn(II)_2_Cys_6_ fungal-type DNA-binding domain (IPR001138) and a nuclear localisation signal (NLS; SHKKSR). Fhs1 homologues are highly conserved in the Pezizomycotina subdivision of Ascomycota, particularly in the class Sordariomycetes, including *Neurospora crassa*, *Podospora anserina*, and *Magnaporthe oryzae* (Supplementary Fig. S1a,b). No homologue has been functionally characterised to date.

### Effect of HU on *fhs1* mutants

**Figure 2:**
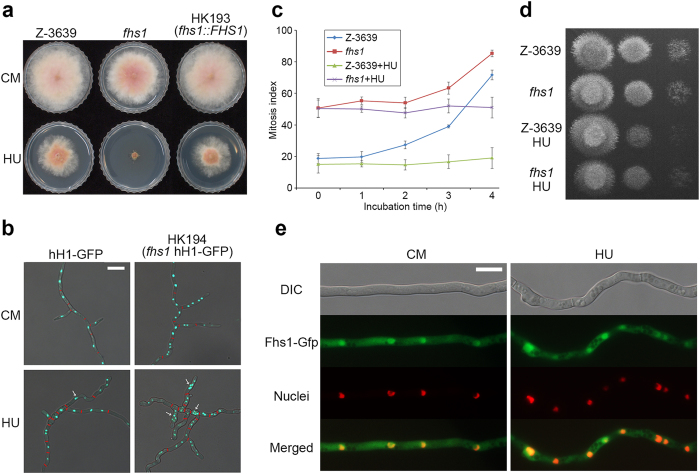
Fungal specific transcription factor Fhs1 involved in hydroxyurea sensitivity. (**a**) Mycelia growth of *F. graminearum* strains on CM and CM supplemented with 10 mM HU. Pictures were taken 4 days after inoculation. (**b**) Nuclei and septa formation in hyphae. Conidia of both strains were inoculated into CM and CM supplemented with 10 mM HU for 24 h. Histone H1 was tagged with Gfp to visualise nuclei in both wild-type and *fhs1* mutant strains. Septa confirmed by Calcofluor white staining are indicated with red bars. DIC and fluorescent protein images were merged. Scale bar = 20 μm. (**c**) Mitosis assay. Germlings bearing cells with two or more nuclei for 4-h incubation with and without 100 mM HU were scored. One hundred conidia were assessed for each strain with three biological replicates. (**d**) Serial dilutions of all strains were point-inoculated onto CM after 4-h incubation with or without HU treatment (10^6^, 10^5^, and 10^4^ conidia/ml). (**e**) Subcellular localisation of Fhs1. Fhs1 was fused with Gfp, and histone H1 was fused with Rfp. Mycelia were harvested for microscopic observation 12 h after conidia inoculation in liquid CM supplemented with or without 5 mM HU. The yellow colour in the merged images indicates colocalisation. DIC, differential interference contrast. Scale bar = 10 μm.

To confirm the function of *FHS1*, targeted gene deletion and complementation experiments were performed. Both genetic manipulations were confirmed using Southern blot analyses (Supplementary Fig. S1c,d). The *fhs1* deletion mutant exhibited a slight growth defect on CM compared with wild-type and was highly sensitive to HU supplementation in accordance with our screening data (Fig. 2a). The complemented strain fully abolished the high HU sensitivity of the *fhs1* mutant, confirming the role of *FHS1* in HU resistance.

To visualise the nuclei of hyphae, the HK194 strain (Δ*fhs1 hH1-GFP*) was generated from an outcross between the mat1g (Δ*mat1 hH1-GFP*) and *fhs1* strains (Supplementary Table S2). Septa visualised using Calcofluor white staining were marked with red bars. Compared with the strain hH1-GFP carrying an *FHS1* wild-type allele, the *fhs1* mutant often produced meandering hyphae (Fig. 2b). Moreover, each hyphal cell of the *fhs1* mutant tended to contain more nuclei (2.9 nuclei per cell) compared with the hH1-GFP strain (1.4 nuclei per cell). When HU was exogenously amended, hyphae of the hH1-GFP strain with wild-type *FHS1* began to meander and the number of nuclei per cell increased as in the *fhs1* mutant without HU supplementation (2.6 nuclei per cell). The phenotypic defect became more severe when HU was added to the *fhs1* mutant (4.9 nuclei per cell).

Because the lack of *FHS1* seemed to cause defects in nuclear division (Fig. 2c), we further performed a mitosis assay. Conidia of *fhs1* mutants bore more nuclei than wild type. When conidia were incubated with a high concentration of HU (100 mM), nuclear division was arrested in the wild-type and *fhs1* stains. After 4 h of HU treatment, both wild-type and *fhs1* mutant strains resumed mycelial growth (Fig. 2d).

### Subcellular localisation of Fhs1

To examine Fhs1 localisation, the *FHS1-GFP* fusion construct under the control of its native promoter was transformed for genetic complementation (Supplementary Fig. S1b). To compare Fhs1-Gfp with nuclei, the HK224 (∆*fhs1::FHS1-GFP-HYG hH1-RFP-GEN*) strain was generated from an outcross between the mat1r and HK193 strains. Fhs1-Gfp in the HK224 strain accumulated in the nuclei (Fig. 2e). localisation patterns were similar regardless of the exogenous HU treatment.

### Sexual development and virulence test

**Figure 3:**
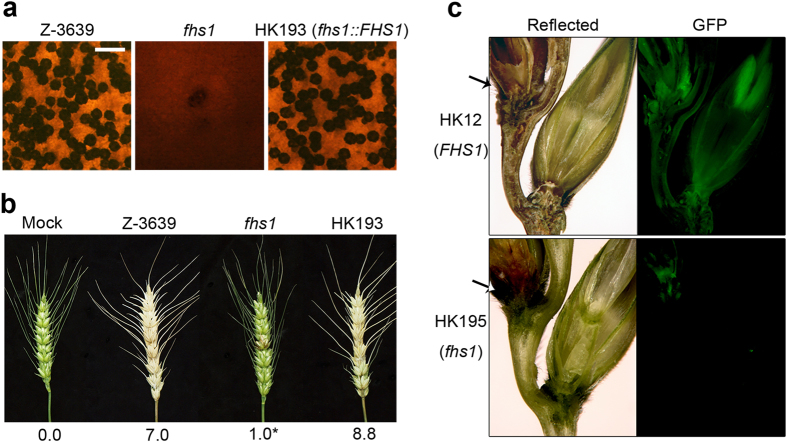
Sexual development and virulence of *F. graminearum* strains. (**a**) Sexual reproduction on carrot agar. Pictures were taken 7 days after sexual induction. Scale bar = 500 μm. (**b**) Virulence on wheat heads. A centre spikelet of each wheat head was injected with 10 μl of conidia suspension. Disease index (diseased spikelets per wheat head) is denoted below the picture. Asterisks indicate that the data differed significantly (*P* < 0.05) based on Tukey′s test. The pictures were taken 14 days after inoculation. Mock, negative control mock-inoculated with 0.01% Tween 20. (**c**) Sections of infected wheat heads. Wheat spikelets were inoculated with conidia suspensions of cytosolic Gfp-expressing *F. graminearum* strains. Infected wheat heads were dissected 6 days after inoculation and examined using fluorescence microscopy. Spreading of the Gfp signal represents spreading of hyphae from the points of inoculation. Arrows mark the inoculated spikelets. Reflected, reflected light.

The wild-type and complemented strains produced normal perithecia bearing mature ascospores 7 days after sexual induction (Fig. 3a). By contrast, vigorous mycelial growth was observed in the sexually induced *fhs1* mutant culture and only a few immature perithecia were produced. Perithecia of the *fhs1* mutant neither matured nor produced ascospores.

The virulence of the fungal strains was examined by point inoculation of wheat spikelets. Fourteen days after inoculation, the wild-type and complemented strains colonised the injected spikelet and adjacent spikelets, causing normal head blight symptoms (Fig. 3b). The *fhs1* mutant infected the inoculated spikelet; however, it lost its ability to spread into the neighbouring spikelets.

To reveal the mechanism underlying the virulence of the *fhs1* deletion mutant, we generated an *fhs1* deletion strain constitutively expressing Gfp to observe mycelial movement during plant infection through an outcross between the KM19 (Δ*mat1 GFP-HYG*) and *fhs1* mutant strains. The resulting HK195 (Δ*fhs1 GFP-HYG*) strain showed the same phenotype as the parental strain *fhs1* mutant and exhibited constitutive expression of cytosolic Gfp. Approximately 6 days after inoculation, the strain (HK12) carrying the wild-type allele of *FHS1* readily colonised the injected spikelet and began to infect adjacent spikelets through rachis nodes (Fig. 3c). The *fhs1* mutant successfully colonised the injected spikelets as in the wild-type, but the hyphae of the *fhs1* mutant could not spread into the adjacent spikelet from the inoculated one. Hyphal growth through rachis was rarely observed in *fhs1* mutants.

### Accumulation of DNA damage in cells

**Figure 4:**
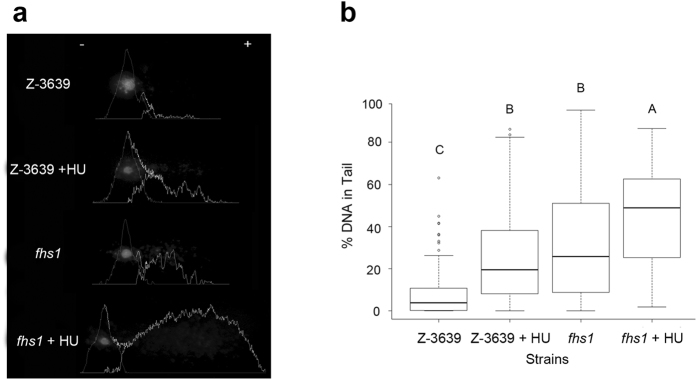
Detection of increased DNA damage. (**a**) Photographs of comets and analysis of the alkaline comet assay. DNA fragments generated from DNA single-strand breaks, double-strand breaks, and alkali-labile sites in the DNA migrate toward the anode, creating the comet assay. Between the comets of *fhs1* and HU-treated wild-type, *fhs1* cells produced longer tails. Graphs depict image analysis quantifying the DNA contents of the head and tail. (**b**) Box-plot of average percentages of DNA in comet tails. *fhs1* cells contained higher percentages of DNA in the comet tail than wild-type but similar to HU-treated wild-type cells (*P* < 0.01). HU-treated *fhs1* cells contained significantly higher percentages of DNA in the comet tail than wild-type and HU-treated wild-type cells (*P* < 0.01).

The alkaline comet assay was performed to analyse DNA single-strand breaks, double-strand breaks, and alkali-labile sites of DNA in each cell. The head of the comet shows undamaged DNA, whereas the tail of comet signals the damaged DNA in each cell. Therefore, cells with more DNA damage exhibit a higher percentage of DNA in the tail. We tested the wild-type and *fhs1* strains with and without HU treatment (Fig. 4). HU induced DNA damage in *F. graminearum*, increasing the comet tail in HU treated cells. DNA damage levels in HU-untreated *fhs1* cells and HU-treated wild-type cells were similar (*P* < 0.01) and both increased relative to the HU-untreated wild-type. This result suggests that deletion of *FHS1* triggered DNA damage in the absence of a DNA-damaging agent. HU-treated *fhs1* cells exhibited significant increases in the percentage of DNA in the tail compared with HU-treated wild-type cells, demonstrating that DNA in *fhs1* mutants is more easily damaged when exposed to HU than the wild-type strain (*P*  < 0.01).

### Transcriptome analyses

To reveal the molecular mechanisms underlying the increased DNA damage level of the *fhs1* mutant, we analysed the transcriptomes of the wild-type and *fhs1* mutant strains using RNA-seq (Supplementary Dataset 1). Genes with transcript levels exhibiting a 3-fold or greater difference between the wild-type and *fhs1* mutant strains were categorised based on their predicted functions (Table 2). Of the total predicted 13,820 genes, 898 (6.5%) were up-regulated and 522 (3.8%) were down-regulated in the *fhs1* strain, respectively. High proportions of genes included in “Cell rescue, defense and virulence”, “Metabolism”, “Cellular transport”, “Interaction with the environment”, and “Energy” were highly up-regulated or down-regulated in the *fhs1* mutant compared with the other groups, except genes encoding “Unclassified proteins”.

**Figure 5:**
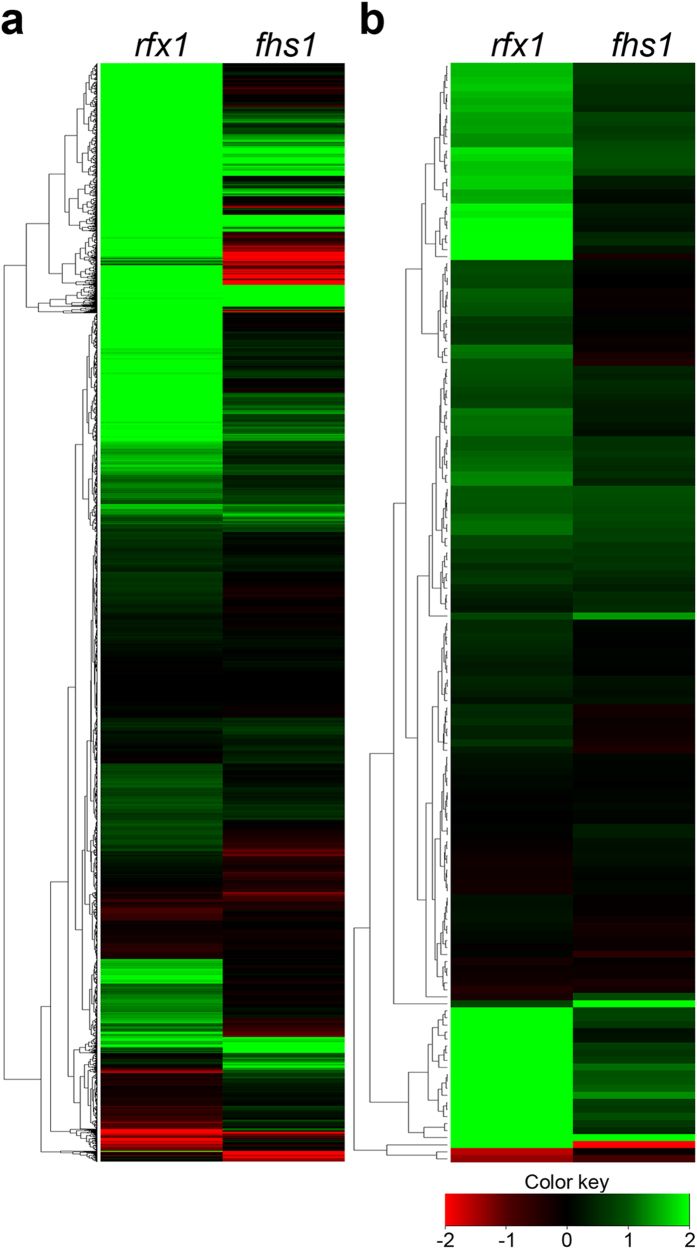
Comparison of the transcriptomes of the *rfx1* and *fhs1* mutants using cluster analysis. (**a**) Heat map depicting the results of cluster analysis of all genes. (**b**) Heat map depicting the results of cluster analysis of genes involved in “DNA repair”. Green represents higher expression, red represents lower expression, and rows represent transcriptional units.

Because *RFX1* deletion also increased DNA damage levels compared with wild-type^12^, we compared the transcriptomes of *rfx1* and *fhs1* mutants (Fig. 5). Approximately one third of total genes (5,300/13,820) were up-regulated and a small portion (146) was down-regulated in the *rfx1* mutant, whereas only 898 and 522 genes were up- and down-regulated, respectively, in the *fhs1* mutant (Fig. 5a). Genes altered in transcript levels were also markedly different between the two mutants. In particular, the transcript levels of DNA repair-related genes were rarely changed in the *fhs1* mutant, whereas 22.4% of these genes (35/156) were up-regulated when *RFX1* was deleted (Fig. 5b). In the *fhs1* mutant, only two genes related to DNA repair (FGSG_05924 and FGSG_16955) were significantly up-regulated (*P*  < 0.01) (Supplementary Dataset 2).

We further examined 18 genes whose homologues in *A. nidulans*^24^ are involved in HU sensitivity to characterise the genetic relationships between *FHS1* and those genes (Supplementary Dataset 3). None of the transcript levels of the homologues including *ATR*, *ATM*, *CHK1*, and *CHK2* were altered in the *fhs1* mutant.

### Characterisation of central regulators for DNA damage responses

To determine whether central regulators of DNA damage responses had functionally conserved roles in *F. graminearum*, we identified *ATM* (FGSG_16493), *ATR* (FGSG_13318), *CHK1* (FGSG_01506), and *CHK2* (FGSG_07121) (Supplementary Dataset 3) and individually deleted genes from the wild-type strain Z-3639 (Supplementary Fig. S2). Outcrossing was used to generate the strains described in Supplementary Table S2. For example, to generate the double deletion mutants, the ∆*mat2* strain was outcrossed with HK210 (*atm* deletion mutant) and HK213 (*chk2* deletion mutant) to select for HK228 (*mat2 atm*) and HK230 (*mat2 chk2*), respectively (Supplementary Table S2). Finally, each double deletion mutant HK231 (*atm atr*) and HK236 (*chk1 chk2*) was obtained from the outcrosses HK228 × HK211 and HK230 × HK212, respectively. HK238 (*fhs1 atr*) and HK239 (*fhs1 chk1*) were generated by transformation due to ascospore lethality.

Three single deletion mutants (*atm*, *atr*, and *chk1*) were more sensitive to HU compared with the wild-type strain. In particular, *atr* and *chk1* mutants rarely grew on CM supplemented with 1 and 2 mM HU, respectively. Both double deletion mutants HK231 (*atm atr*) and HK236 (*chk1 chk2*) exhibited increased sensitivity to HU compared with the corresponding single deletion mutants. Moreover, the double deletion of *ATM* and *CHK1* had a synergistic effect on HU sensitivity. Taken together, we concluded that the cooperative *ATR-CHK1* and *ATM-CHK2* signalling pathways for DNA damage responses induced by HU are conserved in *F. graminearum*.

### Genetic relationships between *FHS1* and central regulators for DNA damage responses

**Figure 6:**
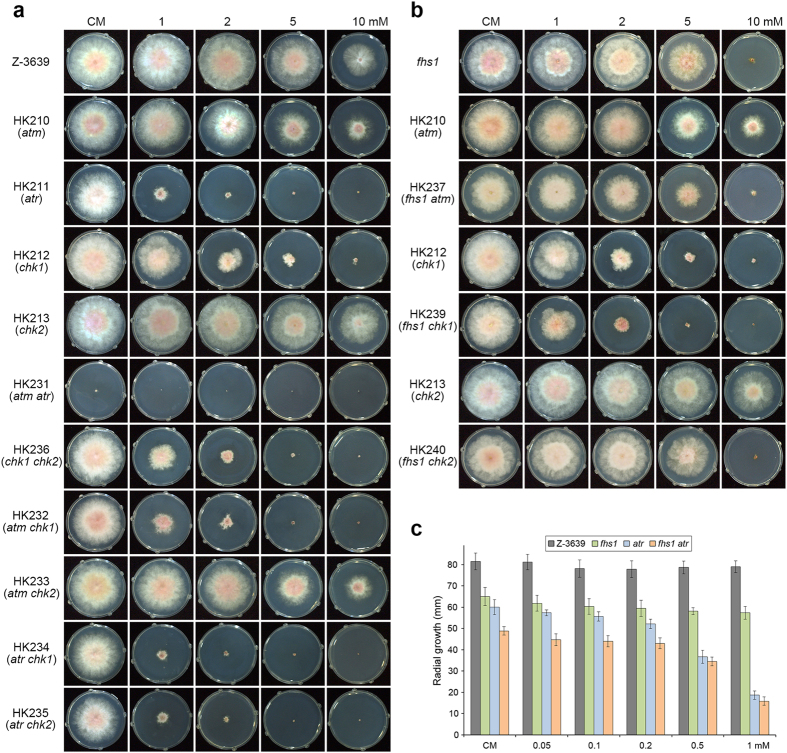
Mycelial growth of *F. graminearum* strains on CM and CM supplemented with HU. (**a**) Mycelial growth of *ATM* and *ATR* pathway gene deletion mutants. (**b**) Mycelial growth of double deletion mutants carrying the *FHS1* deletion. Pictures were taken 3 days after inoculation on CM. (**c**) Radial growth of double deletion mutants carrying the *FHS1* deletion on CM and CM supplemented with 0.05–1 mM HU. Data were obtained 4 days after inoculation.

Radial growth of the double deletion mutants, HK237 (*fhs1 atm*) and HK238 (*fhs1 atr*), both harbouring *FHS1* deletions, was markedly reduced on CM with and without HU supplementation compared with the corresponding single deletion mutants (Fig. 6b,c). And deletion of *FHS1* with *ATM* and *CHK1* exhibited a synergistic effect on HU sensitivity. Transcript levels of *FHS1* were not altered when central regulator genes were deleted under HU treated condition (Supplementary Fig. S3). These results suggest that the *FHS1*-dependent signalling pathway for mitotic cell division and DNA damage response is distinct and independent of central regulators, such as *ATM* and *ATR*.

## Discussion

The increase in applicable molecular tools in *F. graminearum* has enabled large scale genetic approaches and in-depth biochemical studies to dissect the molecular mechanisms underlying fungal development and virulence^25^. The construction of a transcription factor (TF) mutant library shed light on fungal genetics, because TFs are thought to be involved in most biological processes^11^. In addition, chemical genetics utilizing our TF mutant library led us to unveil hidden functions of TFs and new mechanisms for specific cellular processes. A novel regulatory mechanism for sodium tolerance was recently reported from a genetic screen of sodium/lithium-sensitive TF mutants^26^. In the current study, we successfully identified 16 TFs involved in DNA damage responses through genetic screens using four DNA-damaging agents. Further in-depth functional analyses revealed that the novel gene *FHS1* is involved in mitotic cell cycle arrest and DNA damage response in *F. graminearum*.

The results of genetic screens generated in this study provide new insight into the DNA damage responses in filamentous fungi, including *F. graminearum*. We identified that twelve genes (homologues for *ARS2*, *MSN2*, *SFP1*, *DBP3*, *DBP4*, *CTR9*, *CUL-4A*, *RFX1*, *MYO1*, *RAD18*, and *PPR1*) were involved in DNA damage responses, as was previously shown in model organisms, demonstrating that their roles are also conserved in *F. graminearum* and possibly in other filamentous fungi (Table 1). SreA (the homologue of FGSG_09565) is a repressor of siderophore biosynthesis, which is required for iron uptake in *A. nidulans*^23^. Because intracellular iron levels may result in DNA damage^27^, further genetic studies on the role of SreA in DNA damage response are necessary. Moreover, we discovered three novel genes involved in DNA damage responses, two of which (FGSG_01176 and FGSG_00404) are fungal specific TFs included in the Zn(II)_2_Cys_6_ family. Unveiling the up- and downstream pathways for these genes will provide novel fungal specific molecular mechanisms for DNA damage responses.

HU is a specific inhibitor of ribonucleotide reductase (RNR), which is responsible for the synthesis of dNTPs, and therefore stalls DNA replication^28^. Defective cell cycle processes and checkpoint regulation results in hypersensitivity to HU^29^. *FHS1* deletion also resulted in hypersensitivity to HU. Mitosis and alkaline comet assays showed that *FHS1* deletion resulted in defects in the mitotic cell division and subsequent genomic instability (Figs 2c and 4). Moreover, *fhs1* mutants did not exhibit normal DNA damage responses against accumulated DNA damages (Supplementary Dataset 2 and Fig. 5). DNA repair defect of *fhs1* mutant seems to be reason for increased sensitivity to HU than a checkpoint defect since HU successfully arrested mitotic cell division in the wild-type and *fhs1* strains (Fig. 2c). Taken together, we concluded that *FHS1* is required for proper mitotic cell division and DNA damage responses.

Two phosphoinositide 3-kinase-related kinases, Atm and Atr, are highly conserved key factors for cell cycle regulation and DNA damage responses in eukaryotic cells. The downstream effectors Chk1 and Chk2 are directly phosphorylated by Atm and Atr, respectively, for further signal transduction^1^. Whereas the ATM pathway mainly responds to DNA damage, such as double-strand breaks, Atr regulates cell cycle and genes required for DNA damage response caused by disturbed replication forks, such as with HU treatment^30^,^31^. Because our genetic evidence revealed that *FHS1* is required for mitotic cell division and DNA damage responses, we tried to characterise genetic relationships among *ATM*, *ATR*, *CHK1*, *CHK2*, and *FHS1* in *F. graminearum* (Fig. 6). Our genetic evidence revealed that the *ATR-CHK1* and *ATM-CHK2* pathways and their cross talk are conserved in *F. graminearum*. However, *FHS1*-associated regulatory mechanisms appear to be distinct from and independent of both ATR and ATM pathways. We also could not find distinct genetic correlation between *FHS1* and 14 homologous genes responsible for *A. nidulans* S-phase checkpoint mutations (Supplementary Dataset 3).

Hypersensitivity to HU and fungal development of double mutations between *FHS1* and *ATR* were synergistic. Defects in vegetative growth and HU sensitivity were markedly increased in the *fhs1 atr* double deletion mutants compared with single deletion mutants (Fig. 6b,c). Moreover, outcrosses between *fhs1* and *atr* or *chk1* to generate double deletion mutants resulted in ascospore lethality, suggesting that they have overlapping roles in fertility. Taken together, the biological roles of the *FHS1*-dependent pathway and the ATR pathway for DNA damage response and fungal development are not wholly distinct.

Various fungal developmental processes, including sexual and asexual reproduction and virulence, necessarily involve the reprograming of cell cycle regulation for unique morphogenesis. *F. graminearum* produces both sexual (ascospores) and asexual (conidia) spores. Ascospores are produced and forcibly discharged from the perithecia for primary inocula^32^. Perithecia production is a complex cellular differentiation process under polygenic control^33^. Conidia function as secondary inocula and are produced from specialized conidiogenous cells^34^. Moreover, the fungus differentiates hyphal cells to appressoria-like structures for successful plant infection^35^. Therefore, disruption of the cell cycle usually results in severe pleiotropic defects in *F. graminearum*^12^. In addition, accumulation of DNA damages triggered by plant defense responses such as increased levels of reactive oxygen species or reactive nitrogen species might be the reason for avirulent feature of *fhs1* mutant^36^. Fhs1-dependent regulations for mitosis and DNA damage responses are specifically related to sexual development and virulence but not closely related to asexual development or vegetative growth.

In summary, we identified 16 TF genes involved in cell cycle regulation and/or DNA damage responses in *F. graminearum*. *FHS1* is related to DNA damage response and genome integrity in *F. graminearum*. Moreover, disruption of *FHS1* function resulted in DNA damage in cells and multiple defects in virulence and sexual development. Because *FHS1* participates in novel signalling pathways for cell cycle regulation and DNA damage response, further study will focus on the characterisation of regulons of Fhs1 using chromatin immunoprecipitation-sequencing coupled with transcriptome analysis.

## Methods

### Fungal strains and media

The wild-type strain Z-3639 and transgenic strains derived from this strain were used in this study (Supplementary Table S2). *F. graminearum* transcription factor gene deletion mutants were obtained from a previous study^11^. All strains were stored as conidia and mycelia in 30% glycerol solution at −80 °C. Conidia production was induced in carboxymethyl cellulose medium (CMC) or on yeast malt agar (YMA) as previously described^37^,^38^. All of the other media used in this study were prepared as described in the *Fusarium* laboratory manual^32^.

The DNA-damaging agents used in the study were MMS, HU, BLM, and CPT. The concentration of each DNA-damaging agent at which the wild-type strain exhibited approximately half of the radial growth observed on CM without any DNA-damaging agent was established (Supplementary Table S1). CM supplemented with each DNA-damaging agent was used to screen 657 transcription factor gene deletion mutants. Statistical and clustering analyses were conducted using *R* statistical software packages^39^.

### Nucleic acid manipulation, PCR primers and conditions

Genomic DNA was extracted from lyophilised mycelia according to the *Fusarium* laboratory manual^32^. Restriction endonuclease digestion, agarose gel electrophoresis, Southern blotting, and hybridisation with ^32^P-labeled probes were performed following standard protocols^40^. Total RNA was isolated from mycelia ground in liquid nitrogen using the Easy-Spin Total RNA Extraction Kit (iNtRON Biotech, Seongnam, Korea).

PCR and quantitative real-time (qRT)-PCR primers used in this study were synthesised by an oligonucleotide synthesis facility (Bionics, Seoul, Korea) (Supplementary Table S3). General PCR procedures were performed in accordance with the manufacturer’s instructions (TaKaRa Bio Inc., Otsu, Japan).

### Genetic manipulations and fungal transformations

The double-joint (DJ) PCR strategy was applied to construct fusion PCR products for targeted gene deletion and complementation^41^. For *FHS1* deletion, the 5′ and 3′ flanking regions of *FHS1* were amplified from the genomic DNA of the wild-type strain using the primer pairs FHS1-5F/FHS1-5R and FHS1-3F/FHS1-3R, respectively. A geneticin resistance gene cassette (*GEN*) under the control of the *A. nidulans trpC* promoter and terminator was amplified from pII99 using a primer pair Gen-for/Gen-Rev. Three amplicons (5′ flanking region, *GEN*, and 3′ flanking region) were mixed at 1:3:1 molar ratio and fused by a second round of DJ PCR. Finally, the fusion constructs were amplified with the nested primers using the second round PCR product as template.

For complementation, the 5′ flanking region that included the *FHS1* open reading frame with its own promoter and 3′ flanking region were amplified from genomic DNA of the wild-type strain using the primer pairs FHS1-5F/FHS1-5R GFP and FHS1-3F GFP/FHS1-3R, respectively. The *GFP-HYG* construct was amplified from pIGPAPA using pIGPAPA-sGFP/HYG-F1 primers. Three amplicons were then fused in a second round of DJ PCR. Finally, the fusion constructs for transformation were amplified with the nested primers using the second round PCR product as a template.

Fungal transformation was performed as previously described^42^. Conidia were harvested 3–4 days after inoculation in CMC medium, and freshly harvested conidia were inoculated into 50 ml YPG (1% yeast extract, 1% peptone, and 2% glucose) for 12 h at 25 °C. To generate protoplasts, mycelia were harvested through sterilised filter paper and incubated in 35 ml NH_4_Cl containing 10 mg/ml Driselase (Sigma-Aldrich, St. Louis, MO, USA) for 3 h at 30 °C with shaking (50 rpm). Polyethylene glycol (PEG)-mediated fungal transformation was applied for deletion and complementation. Fungal transformants carrying *GEN* or *HYG* were regenerated in the regeneration medium and overlaid with 1% water agar containing geneticin (150 μg/ml; Sigma-Aldrich, St. Louis, MO, USA) or hygromycin B (150 μg/ml; Calbiochem, La Jolla, CA, USA), respectively. After 2–5 days incubation, antibiotic-resistant colonies were selected for further study.

### Sexual crosses and virulence tests

For selfing, cultures were grown on carrot agar plates for 5 days. Sexual reproduction was induced by removing aerial mycelia with sterile 2.5% Tween 60 solution^32^. For outcrosses, heterothallic ∆*mat1* strain was fertilised with 1 ml of a conidial suspension (10^5^ conidia/ml) from fertilising parents. All cultures were incubated at 25 °C for perithecial development for an additional 10 days under near UV light (wavelength: 365 nm, HKiv Import & Export Co., Ltd., Xiamen, China).

For virulence tests, point inoculation on wheat cultivar Eunpamil’s heads was performed as previously described^42^. The conidia used for inoculation were harvested from CMC cultures and suspended in 0.01% Tween 20 solution at 10^6^ spores/ml. After inoculation with 10 μl conidia suspension in the middle of the spikelet, inoculated plants were incubated in a high humidity chamber at 25 °C for 3 days and transferred to a greenhouse for an additional 11 days to assess disease symptoms. Five replicated inoculations per strain and three independent mutant strains were used in the experiment.

### Microscopic observation

To observe localisation of Fhs1 and histone H1 (hH1) in the nuclei, the mat1r strain (Δ*mat1::GEN hH1-RFP-GEN*) was fertilised with the HK193 (Δ*fhs1::FHS1-GFP-HYG*) strain. Ascospores carrying both *FHS1-GFP-HYG* and *hH1-RFP-GEN* were selected using antibiotic resistance and confirmed using PCR. Localisation was observed in cultures from CM with and without 5 mM HU supplementation. Chitin staining was conducted by adding Calcofluor white stock solution (10 mg/ml) to mycelia samples on slide glasses. Microscopic observation was performed with a DE/Axio Imager A1 microscope (Carl Zeiss, Oberkochen, Germany) using filter set 38HE (excitation 470/40; emission 525/50) for Gfp, filter set 15 (excitation 546/12; emission 590) for Rfp, and filter set 49 (excitation 356; emission 445/50) for Calcofluor white.

### Quantitative real-time PCR (qRT)-PCR

Freshly harvested conidia were inoculated into 50 ml liquid CM and incubated for 24 h at 25 °C with shaking. Mycelia were then harvested and inoculated into CM or CM supplemented with 10 mM HU for an additional 2 h. Total RNA was isolated from mycelia that were ground in liquid nitrogen using an Easy-Spin total RNA extraction kit (iNtRON Biotech), and each first-strand cDNA was synthesised using SuperScriptIII reverse transcriptase (Invitrogen, Carlsbad, CA, USA). qRT-PCR was performed with SYBR Green Supermix (Bio-Rad, Hercules, CA, USA) and a 7500 real-time PCR system (Applied Biosystems, Foster City, CA, USA) with corresponding primer pairs (Supplementary Table S3). The ubiquitin C-terminal hydrolase gene *UBH* (FGSG_01231.3) was used as a reference gene. We compared the cycle threshold (2^−∆∆*CT*^) to measure the transcript levels of target genes in different conditions. PCR was performed three times with two replicates per run.

### Alkaline comet assay

The alkaline comet assay was performed as previously described with some modifications^43^. Freshly harvested conidia were incubated in 50 ml YPG at 25 °C for 12 h with shaking. Mycelia were harvested through sterilised filter paper and incubated in 35 ml of 1 M NH_4_Cl containing 10 mg/ml Driselase with or without 10 mM HU supplementation at 30 °C to generate protoplasts. Protoplasts were collected by centrifugation after 4 h and resuspended in 1 M NH_4_Cl (2 × 10^4^ cells/ml).

Low-gelling-temperature agarose (1%, Sigma-Aldrich) was melted in 1 M NH_4_Cl and kept at 40 °C. To improve agarose adhesion, we scored the edges of dust-free frosted-end microscope slides using a diamond-tipped pen. To prepare agarose-precoated slides, the slides were dipped into molten 1% agarose, and one side was wiped clean and air-dried. Protoplast suspension (0.4 ml) was mixed with 1.2 ml low-gelling-temperature agarose at 40 °C in a 5 ml plastic disposable tube. Then, 1.2 ml agarose mixtures were poured onto a pre-coated slide and allowed to gel. Slides were submerged in alkaline lysis conditions to detect DNA single-strand breaks, double-strand breaks, and alkali-labile sites in the DNA, in accordance with a standard protocol^43^. After overnight lysis in the dark, the slides were washed three times with alkaline rinse solution for 20 min each to ensure removal of salt and detergent. Each slide was submerged in fresh alkaline rinse solution in an electrophoresis chamber and electrophoresed for 25 min at a voltage of 0.6 V/cm. Slides were washed with distilled water and stained in ethidium bromide solution, rinsed again with distilled water, and dried at room temperature. The dried agarose was rehydrated for fluorescence microscopy. The pictures for comet analysis were taken on an Axio Imager A1 microscope with a CCD camera and the 550-nm/605-nm filter set. The percentage of DNA in the tail was analysed for individual cells using the CometScore image analysis software (TriTek Corp., Sumerduck, VA).

### RNA-seq analysis

RNA-seq analysis was performed as previously described^34^. Wild-type and *fhs1* conidia were harvested from CMC cultures and inoculated into CM at 25 °C for 24 h with shaking. Total RNA was extracted as stated above. RNA-seq libraries were constructed using the Illumina TruSeq^TM^ RNA sample prep kit with no modifications to the standard low-throughput protocol. Samples were run on an Illumina HiSeq2000 instrument using the reagents provided in the Illumina TruSeq paired-end (PE) cluster kit V3-cBot-HS and the TruSeq SBS kit v3-HS (200 cycles).

The data discussed in this publication have been deposited in NCBI’s Gene Expression Omnibus^44^ and are accessible through GEO Series accession number GSE73891 (http://www.ncbi.nlm.nih.gov/geo/query/acc.cgi?acc=GSE73891). Genome-wide transcript levels were quantified in reads per kilobase of exon per million mapped sequence reads (RPKM)^45^. RPKM values of 0 were changed to 1 to calculate the fold change of the transcript level. Genes for which differential transcript levels were detected were functionally characterised using the Munich Information Centre for Protein Sequences FunCat functional classification and annotation system^46^. Three replicates of RNA-seq were performed for each sample.

## Additional Information

**How to cite this article**: Son, H. *et al.* A novel transcription factor gene *FHS1* is involved in the DNA damage response in *Fusarium graminearum*. *Sci. Rep.* **6**, 21572; doi: 10.1038/srep21572 (2016).

## Supplementary Material

Supplementary Information

Supplementary Dataset 1

Supplementary Dataset 2

Supplementary Dataset 3

## Figures and Tables

**Table 1:** Putative transcription factors involved in DNA damage responses.

Locus ID	DNA-damaging agents				Description of the gene product	Species	Homologue	Reference
	MMS	HU	BLM	CPT				
FGSG_01106		S	S		Related to arsenite-resistance protein 2	Human	*ARS2*	13
FGSG_09368		S		S	Related to C2H2 zinc finger protein	*S. cerevisiae*	*MSN2*	14
FGSG_15767	S	S	S	S	Related to zinc finger protein SFP1	*S. cerevisiae*	*SFP1*	15
FGSG_09565	S	R			Probable siderophore regulation protein (GATA factor)	*A. nidulans*	*SREA*	23
FGSG_01182	S	S	S	S	Conserved hypothetical protein	*S. cerevisiae*	*DBP4*	20
FGSG_05304	S	S	S	S	Conserved hypothetical protein	*S. cerevisiae*	*DBP3*	21
FGSG_01488	S	S			Conserved hypothetical protein	N/A	N/A	N/A
FGSG_06948	S	S			Related to tetratricopeptide repeat protein tpr1	*S. cerevisiae*	*CTR9*	16
FGSG_05520	S	S	S	S	Related to cullin homologue 4A	Human	*CUL-4A*	17
FGSG_07420	S	S	S	S	Related to cephalosporin C regulator 1 (*cpcR1* gene)	*F. graminearum*	*RFX1*	12
FGSG_16982			S	S	Related to myosin 2	*S. cerevisiae*	*MYO1*	18
FGSG_04258	S				Probable DNA repair protein UVS-2	*S. cerevisiae*	*RAD18*	19
FGSG_01176		S			Conserved hypothetical protein	*F. graminearum*	*FHS1*	This study
FGSG_00404	R	R	S		Conserved hypothetical protein	N/A	N/A	N/A
FGSG_00574			S		Related to purine utilisation positive regulator	*S. cerevisiae*	*PPR1*	47
FGSG_00573			S		Related to GAL4-like transcriptional activator	*S. cerevisiae*	*PPR1*	47

MMS, methyl methanesulfonate; HU, hydroxyurea; BLM, bleomycin; CPT, camptothecin; S, more sensitive than wild-type; R, more resistant than wild-type; N/A, not applicable.

**Table 2:** Changes in transcription levels between *fhs1* and wild-type strains by functional category.

FunCat ID	FunCat category name	No. of genes inthe genome	No. of genes up-regulated over 3-fold	No. of genes down-regulated over 3-fold
01	Metabolism	2322	121 (5.21%)	79 (3.40%)
02	Energy	503	22 (4.37%)	15 (2.98%)
10	Cell cycle and DNA procession	659	6 (0.91%)	3 (0.46%)
10.01.05.01	DNA repair	156	2 (1.28%)	1 (0.64%)
11	Transcription	718	4 (0.56%)	6 (0.84%)
12	Protein synthesis	370	0 (0.00%)	3 (0.81%)
14	Protein fate	920	15 (1.63%)	7 (0.76%)
16	Protein with binding function or cofactor requirement	1714	53 (3.09%)	33 (1.93%)
18	Regulation of metabolism and protein function	242	2 (0.83%)	1 (0.41%)
20	Cellular transport, transport facilities and transport routes	1390	63 (4.53%)	51 (3.67%)
30	Cellular communication/signal transduction mechanism	312	5 (1.60%)	3 (0.96%)
32	Cell rescue, defence and virulence	856	67 (7.83%)	34 (3.97%)
34	Interaction with the environment	606	27 (4.46%)	24 (3.96%)
40	Cell fate	240	2 (0.83%)	2 (0.83%)
41	Development	55	2 (3.64%))	2 (3.64%)
42	Biogenesis of cellular components	617	18 (2.92%)	7 (1.13%)
99	Unclassified proteins	9074	716 (7.89%)	398 (4.39%)
–	Total	13820	898 (6.50%)	522 (3.78%)
