# Supplementary material for: A novel transcription factor gene FHS1 is involved in the DNA damage response in Fusarium graminearum
Source: Sci Rep. 2016 Feb 18;6:21572. doi: 10.1038/srep21572 (PMC4757864; doi:10.1038/srep21572)
Supplement: Supplementary Information [file srep21572-s1.doc]

**Supplementary information**

**A novel transcription factor gene *FHS1* is involved in DNA damage response in *Fusarium graminearum***

Hokyoung Son, Minmin Fu, Yoonji Lee, Jae Yun Lim, Kyunghun Min, Jin-Cheol Kim, Gyung Ja Choi, and Yin-Won Lee

**Table S1. DNA-damaging agents used in this study.**

| DNA damage agents | Mechanism | Results | Reference |
| --- | --- | --- | --- |
| MMS 0.1 μl/ml  (Methyl methanesulfonate) | Methylates DNA | DNA synthesis defects | [^1^](#_ENREF_1) |
| HU 10 mM  (Hydroxyurea) | Inhibits ribonucleotide reductase (RNR) | S-phase arrest | [^2^](#_ENREF_2) |
| BLM 10 mU/ml  (Bleomycin) | Imitates gamma irradiation | Double-strand DNA breaks | [^3^](#_ENREF_3) |
| CPT 0.4 μM  (Camptothecin) | Locks topoisomerase I covalently onto the DNA | S-phase strand breaks | [^4^](#_ENREF_4) |

**Table S2. *F. graminearum* strains used in this study.**

| Strain | Genotype | Reference or parents |
| --- | --- | --- |
| Z-3639 | *F. graminearum* wild-type | [^5^](#_ENREF_5) |
| *fhs1* | *∆fhs1::GEN* | [^6^](#_ENREF_6) |
| Δ*mat2* | *∆mat1-2::GFP-HYG* | [^7^](#_ENREF_7) |
| mat1g | *∆mat1-1-1::GEN hH1::hH1-GFP-HYG* | [^8^](#_ENREF_8) |
| mat1r | *∆mat1-1-1::GEN hH1::hH1-RFP-HYG* | [^9^](#_ENREF_9) |
| KM19 | *∆mat1-1-1::GEN GFP-HYG* | [^10^](#_ENREF_10) |
| HK12 | *GFP-HYG* | [^11^](#_ENREF_11) |
| HK193 | *∆fhs1::FHS1-GFP-HYG* | *fhs1* |
| HK194 | *∆fhs1::GEN hH1::hH1-GFP-HYG* | mat1g × *fhs1* |
| HK195 | *∆fhs1::GEN GFP-HYG* | KM19 × *fhs1* |
| HK224 | *∆fhs1::FHS1-GFP-HYG; hH1::hH1-RFP-GEN* | mat1r × HK193 |
| HK210 | Δ*atm::GEN* | Z-3639 |
| HK211 | Δ*atr::GEN* | Z-3639 |
| HK212 | Δ*chk1::GEN* | Z-3639 |
| HK213 | Δ*chk2::GEN* | Z-3639 |
| HK228 | Δ*mat1-2::GFP-HYG* Δ*atm::GEN* | Δ*mat2* × HK210 |
| HK229 | Δ*mat1-2::GFP-HYG* Δ*chk1::GEN* | Δ*mat2* × HK212 |
| HK230 | Δ*mat1-2::GFP-HYG* Δ*chk2::GEN* | Δ*mat2* × HK213 |
| HK231 | Δ*atm::GEN* Δ*atr::GEN* | HK228 × HK211 |
| HK232 | Δ*atm::GEN* Δ*chk1::GEN* | HK229 × HK210 |
| HK233 | Δ*atm::GEN* Δ*chk2::GEN* | HK230 × HK210 |
| HK234 | Δ*atr::GEN* Δ*chk1::GEN* | HK229 × HK211 |
| HK235 | Δ*atr::GEN* Δ*chk2::GEN* | HK230 × HK211 |
| HK236 | Δ*chk1::GEN* Δ*chk2::GEN* | HK230 × HK212 |
| HK237 | Δ*fhs1::GEN* Δ*atm::GEN* | HK228 × *fhs1* |
| HK238 | Δ*fhs1::GEN* Δ*atr::GEN* | HK211 |
| HK239 | Δ*fhs1::GEN* Δ*chk1::GEN* | HK212 |
| HK240 | Δ*fhs1::GEN* Δ*chk2::GEN* | HK230 × *fhs1* |

**Table S3. Primers used in this study.**

| **Primer** | **Sequence (5’ → 3’)** | **Description** |
| --- | --- | --- |
| FHS1-5F | TGGACTCATCGGGCACTAACAAT | Forward and reverse primers for amplification of 5’ flanking region of *FHS1* with tail for the geneticin resistance gene cassette fusion |
| FHS1-5R | gcacaggtacacttgtttagagAGTTGGAAACAGGAGAAAGAAACAGAA |
| FHS1-3F | ccttcaatatcatcttctgtcgCTCATTGTGCCTGATTCCCCC | Forward and reverse primers for amplification of 3’ flanking region of *FHS1* with tail for geneticin resistance gene cassette fusion |
| FHS1-3R | AAGAAGCGTCCCCATACTCTAACACT |
| FHS1-5N | ATTGGACACCACTGTCCTGTTTCCCTA | Forward and reverse nest primers for third fusion PCR for amplification of *FHS1* deletion construct |
| FHS1-3N | TTGCCAAGAGCATGTTTCTGGACTTT |
| Gen-for | CGACAGAAGATGATATTGAAGG | Forward and reverse primers for amplification of the geneticin cassette from the pII99 vector |
| Gen-rev | CTCTAAACAAGTGTACCTGTG |
| pIGPAPA-sGFP F | GTGAGCAAGGGCGAGGAGCTG | Forward and reverse primers for amplification of the *GFP-HYG* construct from pIGPAPA vector |
| HYG-F1 | GGCTTGGCTGGAGCTAGTGGAGG |
| FHS1-5R GFP | gaacagctcctcgcccttgctcacGTCGGGGGAATCAGGCACAA | Reverse primer for amplification of the 5’ flanking region of *FHS1* with a tail for GFP tagging complementation |
| FHS1-3F GFP | cctccactagctccagccaagccGCAACTGCATGTAGCCACGACG | Forward primer for amplification of the 3’ flanking region of *FHS1* with a tail for GFP tagging complementation |
| ATM-5F | TAGGTACCGTTTGCTTATTCCAGGTC | Forward and reverse primers for amplification of 5’ flanking region of *ATM* with tail for the geneticin resistance gene cassette fusion |
| ATM-5R | gcacaggtacacttgtttagagCACGAAATCATCCCCGTTAGGTC |
| ATM-3F | ccttcaatatcatcttctgtcgTTCTCAAGAGGCTGGCATACACAA | Forward and reverse primers for amplification of 3’ flanking region of *ATM* with tail for geneticin resistance gene cassette fusion |
| ATM-3R | TGCTCAGAGACGCCGTTAGTTGTA |
| ATM-5N | CAATTTCTTGGCACCAGCATAAGG | Forward and reverse nest primers for third fusion PCR for amplification of *ATM* deletion construct |
| ATM-3N | TTAGATGGAATGAGGATAGTTGTTGCG |
| ATR-5F | GAACAGGCAGTGTTGAAGTGAGAGAA | Forward and reverse primers for amplification of 5’ flanking region of *ATR* with tail for the geneticin resistance gene cassette fusion |
| ATR-5R | gcacaggtacacttgtttagagCGGTCAAGGAAAGGATCGTCTAATC |
| ATR-3F | ccttcaatatcatcttctgtcgAGAAGAATGGCGCTGAAGGTGTG | Forward and reverse primers for amplification of 3’ flanking region of *ATR* with tail for geneticin resistance gene cassette fusion |
| ATR-3R | TCTTACGAAACACTCCCAAGATAATGTAC |
| ATR-5N | TGGTCTGAAGCGGAATCGTTACAC | Forward and reverse nest primers for third fusion PCR for amplification of *ATR* deletion construct |
| ATR-3N | CTTTCTTGCTGACGGTAGGAACG |
| CHK1-5F | GAACGCGAAGCAAACTCACGA | Forward and reverse primers for amplification of 5’ flanking region of *CHK1* with tail for the geneticin resistance gene cassette fusion |
| CHK1-5R | gcacaggtacacttgtttagagTCTGACGCACCATGATGAAACTCT |
| CHK1-3F | ccttcaatatcatcttctgtcgAAACTCGCACGATACAAATACTGAGC | Forward and reverse primers for amplification of 3’ flanking region of *CHK1* with tail for geneticin resistance gene cassette fusion |
| CHK1-3R | TTCTCAGCACCAGGCAAATACTACG |
| CHK1-5N | TGCCATGCAATCGTGTAATCCTAAC | Forward and reverse nest primers for third fusion PCR for amplification of *CHK1* deletion construct |
| CHK1-3N | TTGCTCAACTTTCCCGACTACACTG |
| CHK2-5F | AGTACCATTTCCAGTAGCCGAGTTGT | Forward and reverse primers for amplification of 5’ flanking region of *CHK2* with tail for the geneticin resistance gene cassette fusion |
| CHK2-5R | gcacaggtacacttgtttagagCGACACGAGATGGAATGATGAGTG |
| CHK2-3F | ccttcaatatcatcttctgtcgTGACTCGAGCATTTACCCCACAG | Forward and reverse primers for amplification of 3’ flanking region of *CHK2* with tail for geneticin resistance gene cassette fusion |
| CHK2-3R | TCACATCGATAGCCCACATCTCAA |
| CHK2-5N | TGGTAAGGAAGGACGGCAGGTATT | Forward and reverse nest primers for third fusion PCR for amplification of *CHK2* deletion construct |
| CHK2-3N | ATATCGGGGCGTGAACTTACTGCT |


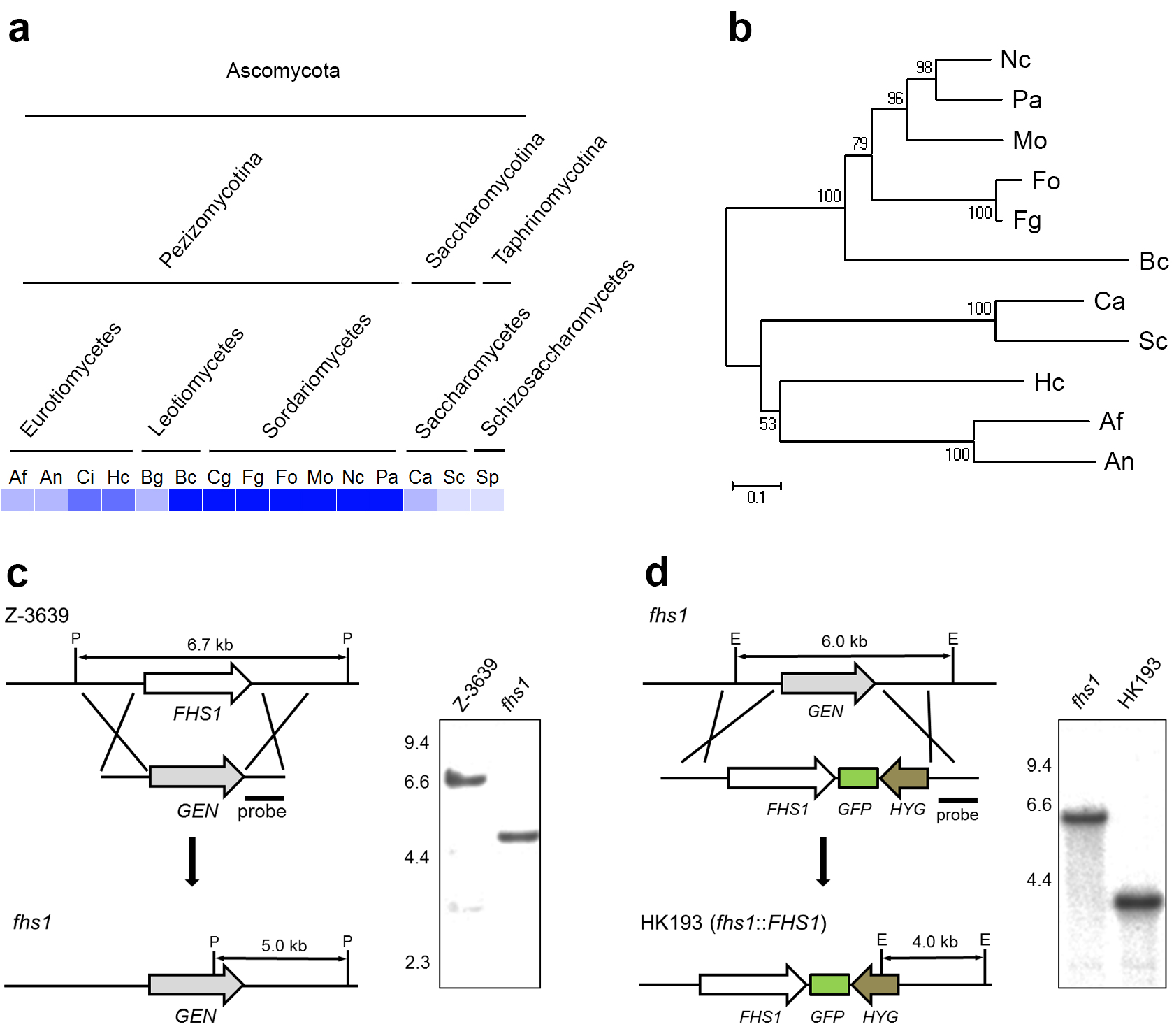


**Figure S1. Characterization and targeted deletion and complementation of *FHS1.*** (**a**) Distribution of Fhs1 homologs in representative fungal species. The distribution image was constructed using the BLASTMatrix tool that is available on the Comparative Fungal Genomics Platform (http://cfgp.riceblast.snu.ac.kr/)[^12^](#_ENREF_12). (**b**) Phylogenetic tree of Fhs1 homologues in several fungal species. ClustalW was used to perform the alignment, and the MEGA program version 4.0 was used to perform a bootstrap phylogenetic analysis using the neighbour joining method. Af: *Aspergillus fumigatus*; An: *A. nidulans*; Ci: *Coccidioides immitis*; Hc: *Histoplasma capsulatum*; Bg: *Blumeria graminis*; Bc: *Botrytis cinerea*; Cg: *Colletotrichum graminicola*; Fg: *Fusarium graminearum*; Fo: *F. oxysporum*; Mo: *Magnaporthe oryzae*; Nc: *Neurospora crassa*; Pa: *Podospora anserina*; Ca: *Candida albicans*; Sc: *Saccharomyces cerevisiae*; Sp: *Schizosaccharomyces pombe*. (**c**) The deletion of *FHS1* was achieved using homologous recombination. Deletion mutants were confirmed by Southern analysis. (**d**) Complementation experiments were performed and the resulting strains were confirmed by Southern analysis. The sizes of the DNA standards (kb) used are indicated to the left of each blot. Z-3639, *F. graminearum* wild-type strain; *fhs1*, *FHS1*-deleted strain; HK193, *fhs1*-derived strain complemented with *FHS1*; *GEN*, genetic resistance gene cassette; *HYG*, hygromycin B resistance gene cassette; *GFP*, green florescence protein gene; P, PstI. E, EcoRV.


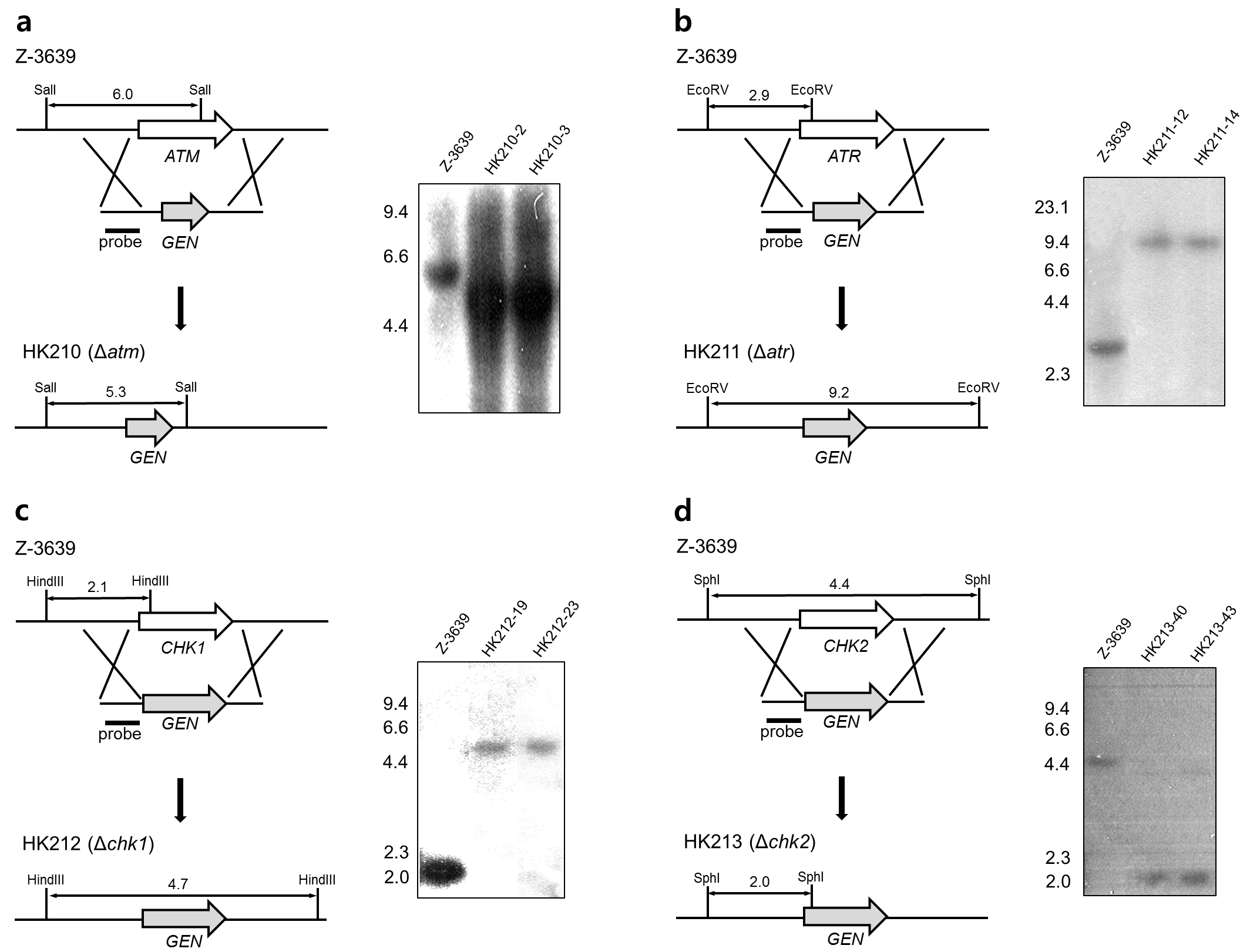


**Figure S2. Targeted gene deletion.** Each *ATM* (**a**), *ATR* (**b**), *CHK1* (**c**), and *CHK2* (**d**) genes were deleted from the genome of *F. graminearum* wild-type strain Z-3639. *GEN*, geneticin resistance gene cassette. The sizes of DNA standards (in kilobases) are indicated on the left of the blot.


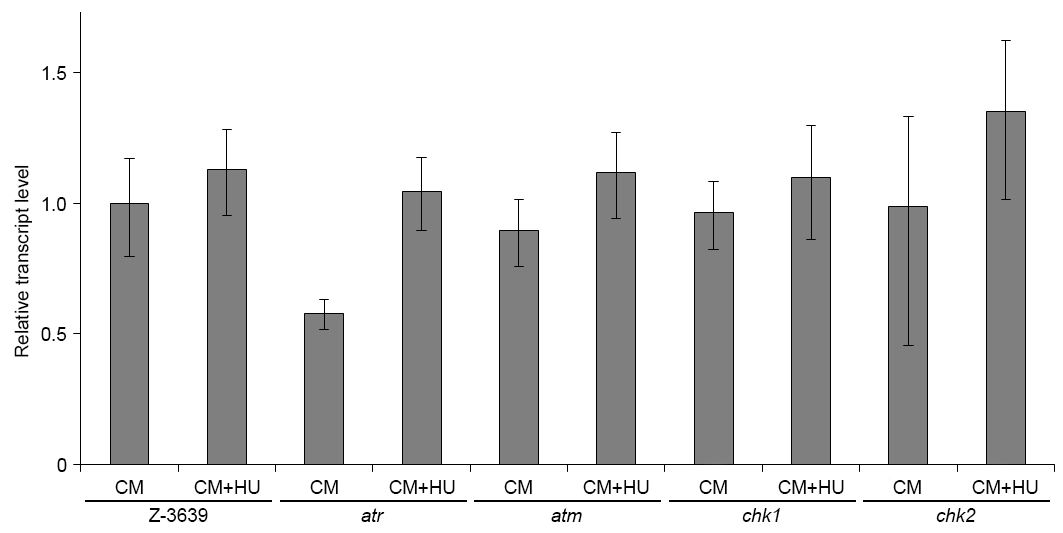


**Figure S3. Relative transcript accumulation of *FHS1* in *F. graminearum* strains.** Transcript levels of *FHS1* were determined in wild-type, *atr* (HK211), *atm* (HK210), *chk1* (HK212), and *chk2* (HK213) with (CM+HU) and without hydroxyurea supplementation (CM) for 0.5 h. The values were generated based on three biological replicates.

**References**

1. Chang M, Bellaoui M, Boone C, Brown GW. A genome-wide screen for methyl methanesulfonate-sensitive mutants reveals genes required for S phase progression in the presence of DNA damage. *Proc Natl Acad Sci U S A* **99**, 16934-16939 (2002).

2. Zhao X, Muller EGD, Rothstein R. A suppressor of two essential checkpoint genes identifies a novel protein that negatively affects dNTP pools. *Mol Cell* **2**, 329-340 (1998).

3. Levin JD, Demple B. *In vitro* detection of endonuclease IV-specific DNA damage formed by bleomycin *in vivo*. *Nucleic Acids Res* **24**, 885-889 (1996).

4. Wan S, Capasso H, Walworth NC. The topoisomerase I poison camptothecin generates a Chk1-dependent DNA damage checkpoint signal in fission yeast. *Yeast* **15**, 821-828 (1999).

5. Bowden RL, Leslie JF. Sexual recombination in *Gibberella zeae*. *Phytopathology* **89**, 182-188 (1999).

6. Son H*, et al.* A phenome-based functional analysis of transcription factors in the cereal head blight fungus, *Fusarium graminearum*. *PLoS Pathog* **7**, e1002310 (2011).

7. Lee J, Lee T, Lee Y-W, Yun S-H, Turgeon BG. Shifting fungal reproductive mode by manipulation of mating type genes: obligatory heterothallism of *Gibberella zeae*. *Mol Microbiol* **50**, 145-152 (2003).

8. Hong S-Y*, et al.* Functional analyses of two syntaxin-like SNARE genes, *GzSYN1* and *GzSYN2*, in the ascomycete *Gibberella zeae*. *Fungal Genet Biol* **47**, 364-372 (2010).

9. Son H, Lee J, Park AR, Lee Y-W. ATP citrate lyase is required for normal sexual and asexual development in *Gibberella zeae*. *Fungal Genet Biol* **48**, 408-417 (2011).

10. Min K, Son H, Lee J, Choi GJ, Kim J-C, Lee Y-W. Peroxisome function is required for virulence and survival of *Fusarium graminearum*. *Mol Plant-Microbe Interact* **25**, 1617-1627 (2012).

11. Son H, Min K, Lee J, Raju NB, Lee Y-W. Meiotic silencing in the homothallic fungus *Gibberella zeae*. *Fungal Biol* **115**, 1290-1302 (2011).

12. Park J*, et al.* CFGP: a web-based, comparative fungal genomics platform. *Nucleic Acids Res* **36**, D562-D571 (2008).
